# Influence of Prenatal Lead Exposure on Genomic Methylation of Cord Blood DNA

**DOI:** 10.1289/ehp.0800497

**Published:** 2009-03-25

**Authors:** J. Richard Pilsner, Howard Hu, Adrienne Ettinger, Brisa N. Sánchez, Robert O. Wright, David Cantonwine, Alicia Lazarus, Héctor Lamadrid-Figueroa, Adriana Mercado-García, Martha Maria Téllez-Rojo, Mauricio Hernández-Avila

**Affiliations:** 1 Department of Epidemiology, University of Michigan School of Public Health, Ann Arbor, Michigan, USA; 2 Robert Wood Johnson Health and Society Scholar, University of Michigan, Ann Arbor, Michigan, USA; 3 Department of Environmental Health Sciences, University of Michigan School of Public Health, Ann Arbor, Michigan, USA; 4 Department of Environmental Health, Harvard School of Public Health, Boston, Massachusetts, USA; 5 Channing Laboratory, Brigham and Women’s Hospital, Harvard Medical School, Boston, Massachusetts, USA; 6 Department of Biostatistics, University of Michigan School of Public Health, Ann Arbor, Michigan, USA; 7 Children’s Hospital, Harvard Medical School, Boston, Massachusetts, USA; 8 Division of Program Evaluation and Biostatistics, Center for Evaluation Research and Surveys, National Institute of Public Health, Cuernavaca, Morelos, Mexico; 9 Division of Environmental Health, Center for Population Health Research, National Institute of Public Health, Mexico City, Mexico; 10 Ministry of Health, Mexico City, Mexico

**Keywords:** blood lead, bone lead, DNA methylation, early life, epigenetics, fetal programming, genomic DNA methylation, intergenerational, lead exposure, life course, Mexico

## Abstract

**Background:**

Fetal lead exposure is associated with adverse pregnancy outcomes and developmental and cognitive deficits; however, the mechanism(s) by which lead-induced toxicity occurs remains unknown. Epigenetic fetal programming via DNA methylation may provide a pathway by which environmental lead exposure can influence disease susceptibility.

**Objective:**

This study was designed to determine whether prenatal lead exposure is associated with alterations in genomic methylation of leukocyte DNA levels from umbilical cord samples.

**Methods:**

We measured genomic DNA methylation, as assessed by Alu and LINE-1 (long interspersed nuclear element-1) methylation via pyrosequencing, on 103 umbilical cord blood samples from the biorepository of the Early Life Exposures in Mexico to Environmental Toxicants (ELEMENT) study group. Prenatal lead exposure had been assessed by measuring maternal bone lead levels at the mid-tibial shaft and the patella using a spot-source ^109^Cd K-shell X-ray fluorescence instrument.

**Results:**

We found an inverse dose–response relationship in which quartiles of patella lead correlated with cord LINE-1 methylation (*p* for trend = 0.01) and and tibia lead correlated with Alu methylation (*p* for trend = 0.05). In mixed effects regression models, maternal tibia lead was negatively associated with umbilical cord genomic DNA methylation of Alu (β= −0.027; *p* = 0.01). We found no associations between cord blood lead and cord genomic DNA methylation.

**Conclusions:**

Prenatal lead exposure is inversely associated with genomic DNA methylation in cord blood. These data suggest that the epigenome of the developing fetus can be influenced by maternal cumulative lead burden, which may influence long-term epigenetic programming and disease susceptibility throughout the life course.

As of 2006, an estimated ≥ 275,000 children in the United States continue to have blood lead levels exceeding the U.S. Centers for Disease Control and Prevention (CDC) limit of concern of 10 μg/dL (CDC 2006; Meyer et al. 2003). In developing countries, the prevalence of elevated blood lead levels greatly surpasses U.S. numbers and signifies a public health priority of global magnitude (Toscano and Guilarte 2005). Lead exposure produces a wide spectrum of health outcomes, most notably neurocognitive and behavioral deficits in response to pre- and/or postnatal exposures (Needleman et al. 1990). Lead exposure has also been associated with spontaneous abortions (Borja-Aburto et al. 1999) and other adverse birth outcomes, such as preterm deliveries (Andrews et al. 1994; Jelliffe-Pawlowski et al. 2006) and low birth weight (Bellinger et al. 1991; Gonzalez-Cossio et al. 1997), which are risk factors for adverse health outcomes over the life course (Barker et al. 1989; Rich-Edwards et al. 1997). Recently, blood levels < 10 μg/dL during early childhood have been reported to confer reductions in IQ scores (Canfield et al. 2003; Lanphear et al. 2005), further questioning whether a threshold exists for the adverse consequences of lead exposure. Despite the well-documented clinical manifestations of lead toxicity, the biological mechanisms underlying these adverse health effects are poorly characterized.

Lead has long been known to readily diffuse across the placenta (Barltrop 1968; Goyer 1990) and therefore represents a significant mode of exposure to the developing fetus. Pregnancy and lactation are also associated with a marked increase in maternal bone turnover (Sowers et al. 1993). Consequently, it is now appreciated that prenatal lead exposure can occur not only through current maternal environmental exposures, but also through the mobilization of cumulative maternal bone lead stores during pregnancy and lactation (Gulson et al. 1997, 2003; Tellez-Rojo et al. 2002). Thus, bone lead stores represent an environmental threat not only for women with current exposure, but also for women with elevated lead exposures in the past.

Epidemiologic and experimental studies strongly suggest that environmental events during fetal development produce persistent effects on cellular function, which in turn may influence the trajectory of health events throughout the life course (Barker et al. 1989; Godfrey and Barker 2001). A likely target by which early-life environmental events dictate disease susceptibility is through epigenetic programming. The epigenome is likely highly vulnerable to environmental factors during embryogenesis because of the extensive epigenetic reprogramming that occurs shortly after fertilization. In addition, the high rate of mitosis presents an opening during which the elaborate nature of the DNA methylation and chromatin patterning required for normal tissue development is susceptible to environmental influence (Dolinoy et al. 2007b). DNA methylation is the most widely studied of the known epigenetic marks and plays important roles in transcriptional regulation, X chromosome inactivation, embryonic development, imprinting, suppression of parasitic DNA sequences, and maintenance of genomic stability (Li et al. 1993). In animal models, maternal dietary supplementation (Dolinoy et al. 2006; Waterland and Jirtle 2003) and chemical exposures (Anway et al. 2006; Chang et al. 2006; Dolinoy et al. 2007a) have been shown to modulate DNA methylation patterns that were stable among unexposed groups. To date, no data exist regarding the influence of *in utero* environmental lead exposure on DNA methylation levels in humans.

We conducted a cross-sectional study to determine whether biological markers of pre-natal maternal lead exposure are associated with genomic methylation of leukocyte DNA in umbilical cord samples from a birth cohort in Mexico City.

## Materials and Methods

### Sample population

The Early Life Exposures in Mexico to Environmental Toxicants (ELEMENT) study is a group of sequentially enrolled epidemiologic birth cohort studies with the aim of investigating the influence of cumulative maternal lead burden on fetal and infant development. For the present study, which uses data and biological samples from the first birth cohort, maternal–infant pairs were recruited between 1994 and 1995 from three hospitals in Mexico City (Mexican Social Security Institute, Manuel Gea Gonzalez Hospital, and National Institute of Perinatology), which serve low- to moderate-income populations. Exclusion criteria included factors that could interfere with maternal calcium metabolism; medical conditions that could cause low birth weight (< 2,000 g); logistic reasons that would interfere with data collection (households living outside the metropolitan area); delivering a premature neonate (< 37 weeks) or an infant with an Apgar score at 5 min of ≤ 6; conditions requiring placement in a neonatal intensive care unit; a physician’s diagnosis of multiple fetuses; intention not to breast-feed; preeclampsia; psychiatric, kidney, or cardiac diseases; gestational diabetes; history of repeated urinary infections; family or personal history of kidney stone formation; seizure disorder requiring daily medication; ingestion of corticosteroids; or blood pressure > 140 mmHg systolic or > 90 mmHg diastolic. Of the initial 1,382 mothers who remained eligible, 617 agreed to participate and continued in the birth cohort study. Of these, 412 umbilical cord samples had DNA extracted. For this study, we selected only those samples that had > 10 μg of DNA available to analyze for this initial study of genomic DNA methylation markers LINE-1 (long interspersed nuclear elements-1) and Alu. Our final study population consisted of 103 umbilical cord samples.

The study protocol was approved by the Ethics Committee of the National Institute of Public Health of Mexico, the participating hospitals, the Brigham and Women’s Hospital, the Harvard School of Public Health, and the University of Michigan. All participating mothers received a detailed explanation of the study intent, research procedures, and counseling on how to reduce environmental lead exposure.

### Blood lead measurements

Umbilical cord venous blood samples were collected in trace metal–free tubes at delivery. Blood samples were analyzed using an atomic absorption spectrometry instrument (model 3000; PerkinElmer, Chelmsford, MA, USA) at the metals laboratory of the American British Cowdray Hospital in Mexico City. External blinded quality-control samples were provided throughout the study period by the Maternal and Child Health Bureau and the Wisconsin State Laboratory of Hygiene Cooperative Blood Lead Proficiency Testing Program.

### Bone lead measurements

Maternal bone lead was measured noninvasively around 1 month postpartum using a spot-source ^109^Cd K-shell X-ray fluorescence (K-XRF) instrument constructed at Harvard University and installed in a research facility in the American British Cowdray Medical Center. The physical principles, technical specifications, and validation of this and other similar K-XRF instruments have been described in detail elsewhere (Aro et al. 1994). For this study, 30-min measurements were taken at the mid-shaft of the left tibia (cortical bone) and the left patella (trabecular bone). Analysis of means and standard deviations of phantom-calibrated measurements did not disclose any significant shift in accuracy or precision. As a quality control measure, we excluded any tibia lead measurements with an uncertainty > 10 μg/g or any patella lead measurements with an uncertainty > 15 μg/g.

### DNA extraction and bisulfite conversion

DNA extraction was performed in the Harvard-Partners Center for Genetics and Genomics. We extracted high-molecular-weight DNA with commercially available PureGene Kits (Gentra Systems, Minneapolis, MN, USA) from the white blood cells of archived umbilical cord blood samples that were collected at delivery. After transport to the Environmental Epigenetics Laboratory at the University of Michigan School of Public Health, DNA samples (200 ng at 10 ng/μL) were bisulfite-treated using the EZ-96 DNA Methylation-Gold Kit (Zymo Research, Orange, CA, USA). Bisulfite conversion of DNA changes unmethylated cytosine to uracil and subsequently to thymidine after polymerase chain reaction (PCR), whereas methylated cytosines are protected from bisulfite conversion, resulting in methylation-dependent differences in DNA sequences. Bisulfite-converted DNA was stored at −20°C until further use.

### Quantitation of LINE-1 and Alu methylation

We measured LINE-1 and Alu methylation by quantitative pyrosequencing using primers and conditions as described previously (Bollati et al. 2007; Choi et al. 2007). We performed the LINE-1 assay using 10 pmol of forward primer 5′-TTTTGAGTTAGGTGTGGGATATA-3′ and 10 pmol of reverse biotinylated primer 5′-AAAATCAAAAAATTCCCTTTC-3′ using the following running conditions: 95°C for 14.5 min, then 35 cycles of 30 sec each at 95°C, 55°C, and 72°C, followed by 72°C for 7 min. We performed the Alu assay using 10 pmol of forward biotinylated primer 5′-TTTTTATTAAAAATATAAAAATT-3′ and 10 pmol of reverse primer 5′-CCCAAACTAAAATACAATAA-3′ using the following conditions: 95°C for 14.5 min, then 40 cycles for 30 sec each at 95°C, 42°C, and 72°C, followed by 72°C for 7 min. We performed the Alu and LINE-1 assays in a 25-μL PCR using HotStarTaq Master Mix (Qiagen, Valencia, CA, USA). For sample controls, we used human genomic DNA that had undergone whole-genome amplification to remove CpG methylation and a human methylated standard (Zymo Research) as 0% and 100% methylated controls, respectively.

Pyrosequencing involves use of a primer extension reaction, using a biotin-labeled single-stranded PCR amplicon as template, in which pyrophosphatase is released during the incorporation of each nucleotide in equimolar proportion to that incorporated. Incorporation of either T (for unmethylated cytosine) or C (for methylated cytosine) at each CpG provides a quantitative measure for consecutive CpG sites throughout the region sequenced. We used the biotin-labeled primer to purify the final PCR product using streptavidin Sepharose High Performance beads (Amersham Biosciences, Uppsala, Sweden). Sepharose beads bound to the PCR product were purified, denatured, and washed using the Pyrosequencing Vacuum Prep Tool (Qiagen, Valencia, CA, USA). We annealed pyrosequencing primers (0.3 μmol/L) to the purified PCR product and sequenced them on a PSQ HS96 Pyrosequencing System (Qiagen). Sequencing primers for Alu and LINE-1 were 5′-AATAACTAAAATTACAAAC and 5′-AGTTAGGTGTGGGATATAGT, respectively. We quantified the level of methylation for each CpG target region using the Pyro Q-CpG Software (Qiagen). This software assigns quality scores for each measurement and internal quality controls to assess the efficiency of bisulfite conversion.

### Statistical analyses

We analyzed data using SAS (version 9.1; SAS Institute Inc., Cary, NC, USA) and R 2.6.1 (R Foundation for Statistical Computing, Vienna, Austria). We examined descriptive statistics and identification of outliers using the generalized extreme studentized deviation method (Rosner 1983) for all variables. All cord blood lead measures were log_e_-transformed before statistical analysis. For both LINE-1 and Alu, we averaged the DNA methylation measures across CpG cites within individuals and used the individual averages to construct descriptive statistics of the study sample.

We used mixed-effects regression models to describe the relationships between infant DNA methylation measures of LINE-1 and Alu and biomarkers of lead exposure with and without adjusting for covariates of interest. We used mixed-effects models because three (Alu) or four (LINE-1) measures (at each CpG dinucleotide site) are available per individual. The basic model employed was *y_ij_* = β_0_ + β_1_ lead.measure*_i_* + *b_j_* + ɛ*_ij_*, where *i* indexes the individual, and *j* = 1,2,3 (or *j* = 1,2,3,4) indexes CpG site. In the model, β_0_ represents the population average methylation percentage across CpG sites, whereas the random effect *b_j_* captures departures from the overall mean for each CpG site. The random errors ɛ*_ij_* values capture individual variability at each CpG site and may be correlated within individuals. We considered two covariance structures to model the correlation of observations within individuals: an unstructured covariance and a compound symmetry structure (which is equivalent to modeling an individual-level random intercept). We used likelihood ratio tests to choose the correlation structure for the error term ɛ*_ij_*; for both Alu and LINE-1 the unstructured correlation had significantly better fit (*p* < 0.001).

We estimated models for each marker of lead exposure as well as for bone lead measures adjusting for blood lead concentrations. We treated the lead exposure measures as continuous variables, as a set of indicator variables to represent exposure quartiles, or as ordinal variables representing quartiles for tests of trend. We chose the potential confounding variables considered in our model based on biologic plausibility or those significantly associated with DNA methylation markers or lead exposures (*p* < 0.1: LINE-1 and maternal age, β= −0.09 ± 0.05; tibia lead and infant sex, β= −2.92 ± 1.72) in bivariate analysis; variables included were maternal age at delivery (years), maternal education (years), cigarette smoking during pregnancy (yes/no), and infant sex (male sex as reference group). We also investigated the adjusted relationships between quartiles of lead exposures and DNA methylation levels and used the estimates to construct a graphical representation of the association. We performed regression diagnostics on all models to evaluate multi collinearity and violations of the linear regression model assumptions.

## Results

Table 1 provides demographic and biological characteristics among newborns according to their participation status in the present study from the ELEMENT study. We found no significant differences in mean lead exposures, newborn sex, maternal age, education, smoking status, or body mass index by participation status. Among umbilical cord samples, eight (7.8%) had blood lead concentrations > 10 μg/dL. Table 2 shows the unadjusted associations between quartiles of lead exposures and Alu and LINE-1 methylation. The average methylation of Alu repeats in newborns was 25.76% (range, 22.95–27.75%), whereas LINE-1 methylation was 79.48% (74.15–84.94%). Quartiles of maternal tibia lead levels were inversely associated with Alu methylation in newborn samples (*p* for trend = 0.04), whereas quartiles of maternal patella lead measures were inversely associated with LINE-1 methylation in newborn samples (*p* for trend = 0.007). Moreover, compared with the first quartile, the fourth quartile for maternal patella lead was associated with significantly lower LINE-1 methylation levels in cord samples (β= −1.34, *p* = 0.01). We found no significant associations between cord blood lead measures and Alu or LINE-1 methylation.

Table 3 shows the results from the models adjusting for possible confounding factors such as maternal age, education, smoking status during pregnancy, and newborn sex. Mixed models with controlling for cord blood lead revealed a modest inverse relationship between maternal patella lead and LINE-1 methylation (β= −0.025, *p* = 0.08). We also observed an inverse association between maternal tibia lead and Alu methylation levels with and without controlling for cord blood (β= −0.027, *p* = 0.009, and β= −0.027, *p* = 0.01, respectively). Again, cord blood lead was not significantly associated with DNA methylation.

**Figure 1:**
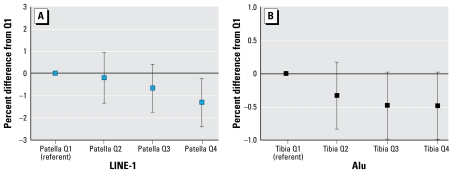
Differences (95% confidence intervals) in percent methylation of cord leukocyte DNA comparing quartiles (Q) of maternal cumulative lead exposure obtained from mixed-effects regression models adjusting for maternal age, education, smoking status during pregnancy, and newborn sex using quartile 1 as referent. (*A*) LINE-1 methylation of cord leukocyte DNA by maternal patella lead quartiles (*p* for trend = 0.01). (*B*) Alu methylation of cord leukocyte DNA by maternal tibia lead quartiles (*p* for trend = 0.05).

Figure 1 illustrates the relationship between quartiles of cumulative lead measures and mean Alu and LINE-1 methylation levels in cord samples after controlling for maternal age, education, smoking during pregnancy, and child sex. An inverse dose–response relationship between patella lead and LINE-1 methylation in cord samples remained significant (*p* for trend = 0.01). Furthermore, quartiles of maternal tibia lead displayed a dose-dependent pattern with Alu methylation in cord samples (*p* for trend = 0.05).

## Discussion

There is increasing epidemiologic and experimental evidence that early-life environmental events affect health outcomes (Barker et al. 1989; Godfrey and Barker 2001). This “fetal origins of disease” hypothesis suggests that environmental factors during development program genetic expression profiles in such a manner that influences the susceptibility to chronic diseases throughout the life course. Unlike the static nature of DNA, the epigenome is relatively dynamic and provides a suitable pathway by which environmental factors can influence disease susceptibility. Although epigenetics have been the center of intense investigation in cancer research, epigenetics has been minimally applied to understand early life events and the impact of environmental risk factors on such events in human populations.

In mammals, DNA methylation almost exclusively occurs within CpG dinucleotides, where an estimated 70% of all CpG dinucleotides are methylated (Robertson and Wolffe 2000). There are two distinct, but seemingly opposing, changes in the epigenome during disease: gene-specific hypermethylation, which is associated with gene repression, and an overall decrease in 5-methyl cytosine content, referred to as genomic hypomethylation. DNA methylation levels in LINE-1 and Alu elements have been routinely used as a surrogate measure of genomic DNA methylation levels (Choi et al. 2007; Perrin et al. 2007; Yang et al. 2004). It is estimated that 0.5 and 1.5 million copies of LINE-1 and Alu exist in the human genome, respectively, and together they comprise around 25% of the genome and > 40% of methylated CpG domains (Choi et al. 2007; Rollins et al. 2006). DNA methylation within these repetitive elements are thought to play a critical role in genomic defense and structural integrity by silencing expression of these transposon elements, thereby limiting chromosomal rearrangement and translocation events (Wilson et al. 2007).

In this study, we examined the associations between maternal lead burden and genomic DNA methylation levels in umbilical cord blood samples. Our most prominent findings were those investigating the impact of maternal cumulative bone lead measures on genomic DNA methylation. In mixed-effects models, maternal tibia and patella lead measures were inversely associated with Alu and LINE-1 methylation levels, respectively. We found no associations between cord blood lead and DNA methylation.

This study, to our knowledge, is the first to examine the effect of maternal lead burden on genomic DNA methylation levels from cord blood samples in humans. Environmental exposures can influence early-life events through two distinct processes, epigenetic fetal programming and transgenerational epigenetic inheritance. Epigenetic fetal programming is limited to modifications of the epigenome of the developing offspring, primarily targeting somatic cells that are responsible for tissue-specific gene expression. Alternatively, environmental factors can induce the transgenerational inheritance of phenotype through germline alterations in the epigenome; however, to be considered a transgenerational effect, these epigenetic changes must persist through the F_3_ generation (Jirtle and Skinner 2007). Previous research using animal models has highlighted the importance of early-life environment events in programming DNA methylation patterns, which in turn alters disease susceptibility throughout the life course. In the agouti mouse model, maternal dietary supplementation, such as methyl-donor groups (Waterland and Jirtle 2003) and genistein (Dolinoy et al. 2006), and maternal bisphenol-A exposure (Dolinoy et al. 2007a) have been shown to alter phenotype and predisposition of offspring to adult-onset obesity and cancers through alterations in DNA methylation during early development. Moreover, brief *in utero* exposure to arsenic has been reported to alter DNA methylation in GC-rich regions among offspring (Xie et al. 2007), producing multiple tissue-specific tumors when the offspring reached adulthood (Waalkes et al. 2003, 2004). Emerging data also suggest that *in utero* exposures to environmental toxicants can elicit epigenetic transgenerational inheritance over multiple generations through effects on the germline (Anway et al. 2006; Chang et al. 2006; Jirtle and Skinner 2007).

Of particular relevance to the impact of lead on fetal origins of disease has been novel work by Zawia and colleagues using rodent and monkey models (Basha et al. 2005; Wu et al. 2008) to demonstrate that early-life lead exposure can predetermine the late-life expression and regulation of the amyloid precursor protein as well as a decrease in DNA methyltransferase activity. These results suggest that epigenetic programming may be a mechanism by which lead alters susceptibility to diseases such as Alzheimer’s disease. Our findings also indicate that the epigenome of the developing fetus is influenced by maternal cumulative lead burden. Taken together, these data suggest that early-life lead exposure may influence long-term epigenetic programming, which in turn may affect susceptibility to disease throughout the life course.

Interestingly, in our study DNA methylation levels were associated only with cumulative measures of bone lead and not with blood lead concentrations. Previous studies from our group have demonstrated that maternal bone lead is a better biomarker than either cord blood or postnatal blood lead for predicting adverse birth outcomes, such as birth weight (Gonzalez-Cossio et al. 1997), and neurodevelopmental outcomes (Gomaa et al. 2002). Lead levels in blood serve as measures of current biologically active lead and are considered a better marker of recent exposures. In contrast, bone lead acts both as a repository for cumulative lead exposure as well as a source of exposure itself upon normal bone turnover and thus is a better indicator of chronic exposure. Therefore, factors that influence bone turnover and thus bone lead mobilization, such as pregnancy and lactation, may modify the toxicity of lead (Gulson et al. 2003; Tellez-Rojo et al. 2002). Indeed, isotopic speciation studies have demonstrated that the skeletal contribution to blood lead levels increases from 9% to 65% during pregnancy (Gulson et al. 1997).

The mechanism(s) involved in lead’s impact on genomic DNA methylation levels in cord samples are unclear. Chronic exposure of lead has been shown to increase the generation of reactive oxygen species (Adonaylo and Oteiza 1999; Antonio-Garcia and Masso-Gonzalez 2008), which have been shown to inhibit binding of methyl-CpG binding proteins and alter DNA methyltransferase function (Valinluck et al. 2004). We can only speculate that perhaps lead-induced oxidative stress may reduce the fidelity of the epigenetic machinery, whereby the developing fetus will be particularly prone to epigenetic errors due to epigenetic reprogramming shortly after implantation and/or the high rate of DNA synthesis. In addition, blood lead has been shown to influence homocysteine levels, which is associated with reductions in DNA methylation (Yi et al. 2000), likely through inhibition of DNA methyltransferases (Cox et al. 1977; James et al. 2002).

The large differences in Alu and LINE-1 methylation levels (26% and 79%, respectively) are assay dependent, because both elements are known to be heavily methylated in normal tissue. The Alu assay, designed based on its consensus sequence, has undergone extensive C-to-T transitions, thereby limiting the pool of CpG sites to be methylated (Yang et al. 2004). The LINE-1 assay, however, targets the promoter region, which is largely 5′-truncated in most LINE-1 elements except those thought to be of more recent evolutionary origin (Estecio et al. 2007; Kazazian 2004).

Given that repeated measures are collected within individuals (at the various CpG cites), we employed mixed effects models as the analysis tool to account for correlation structure of the data. This general approach has been previously used in analyses of DNA methylation (Tarantini et al. 2009). In those analyses and in ours, random effects for CpG site were used to model the average departure of each CpG site from the overall mean (*b_j_*). A random effect for this departure is appropriate because the actual departure perhaps holds little biological meaning (a well-defined biological interpretation could merit these differences be treated as fixed effects). One difference in our approach is that we used an unstructured covariance matrix to model the correlation structure within individuals as opposed to a compound symmetry assumption (equivalent to using a random intercept for individual). Our data supported the use of an unstructured covariance both because the variance in percent methylation varied across sites, and the correlation between pairs of sites was not constant across different pairs of sites. Although the use of random intercepts for individuals lends itself to a nice interpretation (where the random intercept for person represents the individual’s deviation from the overall mean), the unstructured correlation assumption more closely described our data. The issue of selecting a correlation structure is relevant to obtain correct and most efficient standard errors for the regression coefficients, and is perhaps of more relevance for small samples. The need to model the correlation structures correctly can be easily relaxed by using robust standard errors.

A limitation to this study is the use of leukocyte DNA methylation levels as a proxy for lead-induced changes in epigenetic patterns in target tissue for lead toxicity. For example, no data exist regarding whether the extent of methylation levels within circulating DNA are representative to DNA methylation changes in other tissue such as the central nervous system. Furthermore, because leukocyte DNA is a mix of numerous cell types, we cannot rule out the possibility that lead may induce small shifts in white blood cell populations, which may have influenced our findings. An additional limitation to this study is that the LINE-1 and Alu methylation data represent a weighted average of DNA methylation across the genome and does not represent absolute methylation levels, nor does it provide fine epigenomic mapping of DNA methylation patterns across specific chromosomal regions. Additional research is warranted to assess whether prenatal lead exposure influences epigenome-wide DNA methylation patterning as well as gene-specific DNA methylation profiles.

In conclusion, we found an inverse association between cumulative lead measures and genomic DNA methylation among newborn samples. Our results suggest that the epigenome of the developing fetus can be influenced by maternal cumulative lead burdens, suggesting that the epigenome may be a likely target by which intergenerational transmission of lead burdens may influence long-term epigenetic programming, which in turn may affect disease susceptibility throughout the life course.

## Figures and Tables

**Table 1:** Characteristics [mean ± SD or no. (%)] of newborns by participation status.

Measure	Nonparticipating newborns (*n*= 528)	Participating newborns (*n* = 103)	*p*-Value
Maternal tibia lead (μg/g)	9.9 ± 10.4 (*n* = 515)	10.5 ± 8.4 (*n* = 102)	0.27
Maternal patella lead (μg/g)	15.3 ± 15.7 (*n* = 489)	12.9 ± 14.3 (*n* = 100)	0.20
Cord blood lead (μg/dL)	6.6 ± 3.8 (*n* = 417)	6.6 ± 2.7 (*n* = 103)	0.32
Maternal body mass index (kg/m^2^)	23.5 ± 3.4 (*n* = 416)	23.8 ± 4.9 (*n* = 80)	0.61
Maternal age (years)	24.5 ± 5.2	24.4 ± 4.8	0.90
Maternal education (years school)	9.2 ± 4.8	9.5 ± 3.6	0.24
Newborn sex (male)	287 (54.9)	55 (52.9)	0.81
Smoking during pregnancy	24 (4.6)	3 (2.9)	0.45

**Table 2:** Unadjusted Alu and LINE-1 methylation of cord blood (%) by quartiles of lead exposures.

Variable	No.	Alu ± SD	LINE-1 ± SD
Overall mean	103	25.76 ± 1.04	79.48 ± 2.18
Maternal tibia lead (μg/g)			
≤ 4.8	25	26.00 ± 0.94	79.72 ± 2.16
> 4.8 and ≤ 9.7	26	25.78 ± 0.84	79.31 ± 2.53
> 9.7 and ≤ 16.4	26	25.75 ± 1.07	79.55 ± 2.01
> 16.4	25	25.71 ± 1.07	79.37 ± 2.11
*p*-Value for trend^a^		0.04	0.38
Maternal patella lead (μg/g)			
≤ 2.2	25	25.65 ± 0.75	80.08 ± 2.33
> 2.2 and ≤ 12.1	25	26.18 ± 1.06	79.78 ± 1.68
> 12.1 and ≤ 21.8	25	25.84 ± 0.99	79.25 ± 1.91
> 21.8	25	25.52 ± 1.02	78.57 ± 2.48
*p*-Value for trend^a^		0.10	0.007
Cord blood lead (μg/dL)			
≤ 4.4	25	25.68 ± 1.00	79.17 ± 2.09
> 4.4 and ≤ 6.2	27	25.73 ± 1.13	79.37 ± 1.99
> 6.2 and ≤ 7.9	26	25.99 ± 0.77	79.84 ± 2.13
> 7.9	25	25.79 ± 1.00	79.55 ± 2.55
*p*-Value for trend^a^		0.33	0.46

a Obtained using mixed-effects models.

**Table 3:** Mixed-effects regression models of cord blood DNA methylation, and lead biomarkers controlled for maternal age, maternal education, infant sex, and smoking status during pregnancy.

	Mean infant DNA methylation (β ± SE)	
Measure	Alu	LINE-1
Cord blood lead	0.176 ± 0.23	0.45 ± 0.49
Maternal tibia lead	−0.027 ± 0.01**	−0.002 ± 0.02
Maternal tibia lead^a^	−0.027 ± 0.01#	−0.003 ± 0.02
Maternal patella lead	−0.005 ± 0.01	−0.020 ± 0.01
Maternal patella lead^a^	−0.007 ± 0.01	−0.025 ± 0.01*

a Models also controlled for cord blood lead.

* *p* < 0.10.

** *p* < 0.05.

# *p* < 0.01.
